# Association Between Trust in Healthcare Providers and Readiness to Deprescribe in Older Adults: A Cross‐Sectional Comparative Study in the United States and Japan

**DOI:** 10.1002/jgf2.70186

**Published:** 2026-10-07

**Authors:** Kenya Ie, Amy M. Linsky, Xinran Liu, Melina B. Martin, Rie Oyama, Steven M. Albert

**Affiliations:** ^1^ Department of General Internal Medicine St. Marianna University School of Medicine Kawasaki Kanagawa Japan; ^2^ Department of General Internal Medicine Kawasaki Municipal Tama Hospital Kawasaki Kanagawa Japan; ^3^ General Internal Medicine, Center for Health Optimization and Implementation Research (CHOIR), and New England Geriatric Research Education and Clinical Center (GRECC) VA Boston Healthcare System Boston Massachusetts USA; ^4^ General Internal Medicine Boston University Chobanian & Avedisian School of Medicine Boston Massachusetts USA; ^5^ Department of Social Medicine and Health Education Peking University School of Public Health Beijing China; ^6^ Center for Healthy Aging Peking University Health Science Center Beijing China; ^7^ Department of Behavioral and Community Health Sciences University of Pittsburgh School of Public Health Pittsburgh Pennsylvania USA

## Abstract

**Background:**

Understanding how patient trust in healthcare providers is related to readiness to deprescribe is essential for optimizing deprescribing. This study examined this association among older adults with polypharmacy in the U.S. and Japan.

**Methods:**

We conducted cross‐sectional surveys involving Japanese older adults receiving care from a community hospital and primary care clinic (Kawasaki City, *n* = 185) and American older adults recruited to a research registry within a university hospital system (Pittsburgh, *n* = 107). Cohorts were recruited using similar inclusion criteria (5+ prescriptions, community‐resident, stable regimens, recent physician care, no dementia). Participants completed the 11‐item Patient Perceptions of Deprescribing short form to assess readiness to deprescribe (range 6–30) and trust in healthcare providers (range 5–25).

**Results:**

Greater trust in providers was associated with lower readiness to deprescribe across both samples, with no significant interaction by location. A higher number of medications was associated with greater readiness. Although U.S. participants had higher readiness (16.7 vs. 15.4, *p* < 0.05) and trust (21.9 vs. 19.8, *p* < 0.001), the greater readiness observed among U.S. participants remained after adjustment for demographics and clinical characteristics (+2.45 points, *p* < 0.01).

**Conclusions:**

Greater trust in healthcare providers was consistently associated with lower readiness to deprescribe across both samples. Although U.S. older adults demonstrated greater readiness to deprescribe compared to Japanese counterparts, the association between trust and motivation was similar across settings. This finding suggests that how trust functions in patient–provider relationships may be important for designing shared decision‐making interventions for deprescribing.

## 1 Introduction

As populations age, polypharmacy, often defined as regular use of five or more medications, has become a global challenge because of its adverse outcomes, including falls, hospitalization, and reduced quality of life. This challenge is compounded by increasing lifespans without corresponding increases in disability‐free life expectancy, implying more time lived with chronic conditions and greater exposure to medications, and potentially greater risk of adverse events associated with inappropriate prescribing.

In Japan, life expectancy reached 85.2 years in 2021, alongside reduction in mortality from stroke, ischemic heart diseases, and cancers, but with a rising burden of dementia and diabetes [1]. Similarly, in the U.S., where life expectancy in 2021 was 77.1 years, mortality also declined for ischemic heart disease and some cancers, but increased for other causes, such as substance use disorders, chronic kidney disease, and falls [2]. As populations age and multimorbidity becomes more prevalent, polypharmacy is increasingly common among older adults in both Japan and the United States [3, 4].

Deprescribing, the rational and intentional reduction of unnecessary or inappropriate prescriptions conducted by health care professionals in collaboration with patients, is a safe and effective method of reducing harm from polypharmacy. Apart from provider barriers, such as absence of time, multiple providers, and unclear guidelines, patient factors are also important. Successful deprescribing is fostered by a collaborative, multidisciplinary approach and open communication between patients and providers [5]. However, “patients' perspectives toward their medicines have not received sufficient attention in the deprescribing literature,” [6] despite evidence that these perspectives are critical to the success of deprescribing interventions.

Cross‐national studies in Europe have shown large variations in willingness to deprescribe even when patients report comparable satisfaction with medications [7]. These findings highlight the influence of cultural and system‐level factors on patients' readiness to change medications. Similar cultural influences are likely to exist outside Europe as well. Japan and the United States offer a particularly informative contrast given differences between their health systems and norms of patient‐physician relationships. Previous work in Western countries suggests that patient motivation and trust in providers play central roles in whether deprescribing is acceptable and effective, but less is known about these factors in non‐Western countries.

Cultural context may shape these attitudes. In Japan, where deference to physicians remains strong, higher trust might promote adherence but also reduce willingness to question medications [8]. In contrast, in the U.S., where patient autonomy and shared decision‐making are emphasized [9], trust may coexist with greater openness to deprescribing.

The role of cultural context in patient readiness to deprescribe remains unclear. Assessing differences between Asian and North American settings may be valuable for determining shared features of readiness to deprescribe and features that may be specific to a particular setting.

Accordingly, the present study examined the association between trust in healthcare providers and readiness to deprescribe among older adults with polypharmacy in primary care settings in Japan and the United States. Specifically, we examined (i) potential differences in motivation to deprescribe and trust in medical providers among patients in the two settings, (ii) how many differences can be attributed to location rather than patient factors, and (iii) whether the relationship between motivation to deprescribe and trust in providers differs between the two settings.

## 2 Methods

### 2.1 Study Design and Participants

We conducted a cross‐sectional, international comparative study to evaluate the association between trust in healthcare providers and readiness to deprescribe. Older Japanese and American patients 65 years and older with polypharmacy were recruited from patients receiving care at a community hospital (Kawasaki Municipal Tama Hospital, Kawasaki, Kanagawa, affiliated with St. Marianna University School of Medicine; *n* = 166) and a primary care clinic (Tama Family Clinic, Kawasaki, Kanagawa; *n* = 19), and from the University of Pittsburgh's Claude D. Pepper community research registry, which recruits patients receiving care from a university hospital system (*n* = 108). To ensure comparability, inclusion/exclusion criteria were harmonized across sites: age ≥ 65 years, five or more different prescribed medications taken daily (nonprescription and over‐the‐counter medications not included), visit with current primary care physician (or practice) within prior 6 months, no change in medication regimen in prior 4 weeks, and no diagnosis of dementia. Dementia was ascertained differently in the two samples. In the Kawasaki sample, patients with dementia were excluded based on diagnoses listed in medical records. In Pittsburgh, where medical records were not accessed, patients completed the Memory Impairment Screen‐Telephone [10, 11], a brief 8‐point delayed free‐ and cued‐recall test of memory impairment. Participants with scores of 4 or less were excluded.

No formal sample size calculation was performed specifically for the present analysis because this was an exploratory cross‐sectional comparative study using available samples from two settings. Participants with missing baseline SF‐PPoD data were excluded from the analysis, and no imputation procedures were performed.

### 2.2 Measures

All participants completed the short‐form Patient Perceptions of Deprescribing (SF‐PPoD) survey, a brief, validated instrument originally developed in English to assess patients' attitudes toward deprescribing [12, 13]. A culturally adapted and validated Japanese version of the SF‐PPoD was administered [14]. The instrument comprises two subscales: *Motivation to Deprescribe* (6‐items; range 6–30), where higher scores indicate greater willingness to stop unnecessary medications, and *Trust in Healthcare Providers* (5‐items; range 5–25), where higher scores reflect greater trust in providers. Sample items include “I sometimes worry about becoming too dependent on my medicines” (*Motivation to Deprescribe*) and “My PCP makes an effort to listen to the things that matter most to me when it comes to taking medicines” (*Trust in Healthcare Providers*). The two subscales are interpreted separately; no composite score or validated cutoff has been established.

Other measures, including the EQ‐5D‐5L, a measure of health‐related quality of life (https://euroqol.org/), were collected by self‐report. Sociodemographic indicators (including age, sex, and education level), information about prescriptions, experience with deprescribing, diagnosed medical conditions, and health care utilization within the past 6 months were assessed using a structured questionnaire developed by the research team.

### 2.3 Statistical Analyses

Descriptive statistics and *t*‐tests were used to compare characteristics and PPoD scores across the Japanese and American samples. Multiple linear regression was used to examine the association between *Trust in Healthcare Providers* and *Motivation to Deprescribe*, adjusting for location, functional status (EQ‐5D mobility rating), number of prescription medications, age group, sex, and educational attainment (college graduate or higher). Covariates were selected a priori based on previous literature identifying factors potentially associated with attitudes toward deprescribing [5, 15]. Interaction terms were estimated to test whether the relationship between Trust and Motivation to Deprescribe differed across locations. All statistical analyses were conducted using Stata/SE version 17 (StataCorp LLC, College Station, TX, USA). We used InteractionPoweR in R to assess statistical power in testing the interaction term [16].

### 2.4 Use of Artificial Intelligence Tools

OpenAI ChatGPT (GPT‐5.2 and GPT‐5.6 Sol) was used to assist with language editing and proofreading during the preparation and revision of this manuscript. The AI tool was not used for the study design, data analysis, or interpretation of the findings. The authors reviewed and verified all AI‐assisted content and take full responsibility for the final manuscript.

## 3 Results

### 3.1 Enrollment

In the Japanese sample, participants were recruited between May and October 2023. Four hundred and fifty‐nine individuals were invited to participate and 203 responded to the survey (response rate 43.6%). Of these, three were excluded due to missing baseline data and four later withdrew consent. In addition, 11 participants were excluded because of a prior dementia diagnosis, leaving 185 individuals in the final analytic sample.

In the Pittsburgh sample, participants were recruited between October 2023 through June 2024. About a third of people screened from the Pittsburgh Pepper Center Registry (*n* = 205/667) were ineligible because they were taking fewer than 5 prescribed medications or had a recent hospitalization or change in primary care physician. Of 462 potentially eligible registry participants, 117 completed cognitive screening (25.3%), and 107 completed the baseline survey (response rate 23.2%).

### 3.2 Sociodemographic and Clinical Status

The two samples were similar in age (about half aged 75–84) and gender (60.5% male in the Japan sample vs. 46.7% in the U.S. sample, n.s.) (Table 1). More U.S. patients reported college or postcollege years of education (86.9% vs. 36.1%, *p* < 0.001). The two samples were similar in self‐reported health‐related quality of life assessed with the EQ‐5D‐5L. For example, 11.9% of the Japanese sample and 10.3% of the U.S. sample reported fair or poor mobility. The U.S. sample reported significantly more physician‐diagnosed conditions, including cancer, COPD, hypertension, and depression. In contrast, the Japanese sample reported more hospital admissions in the prior 6 months (27%) than American participants (17.4%), a nonsignificant difference (*p* = 0.08), and more prescription medications (mean ± SD) (8.04 ± 2.6 vs. 7.32 ± 2.5, *p* < 0.05, by *t*‐test). In both samples, the most frequently used medications were for chronic cardiovascular and metabolic conditions (e.g., antihypertensives, statins, and antidiabetic agents). Psychotropic medications were also common but differed between the two samples: sedative–hypnotics and anxiolytics were more frequently used in Japan, whereas antidepressants and analgesics were more common in the U.S. (Table S1).

**TABLE 1:** Features of older adults, Kawasaki and Pittsburgh.

	Kawasaki (*n* = 185)	Pittsburgh (*n* = 107)
Male, *n* (%)	112 (60.5)	50 (46.7)
Age, *n* (%)		
65–74	59 (31.9)	45 (42.1)
75–84	98 (53.0)	49 (45.8)
85+	28 (15.1)	13 (12.2)
College graduate^b^, *n* (%)	65 (35.1)	93 (86.9) ***
EQ‐5D^c^, Fair‐Poor, *n* (%)		
Mobility	11 (11.9)	11 (10.3)
Selfcare	1 (0.5)	1 (0.9)
Activities	5 (2.7)	2 (1.9)
Pain	16 (8.7)	10 (9.4)
Depression‐anxiety	4 (2.2)	1 (0.9)
Diagnosed medical conditions, *n* (%)		
Cancer	32 (17.3)	36 (33.6) ***
Hypertension	103 (55.7)	85 (79.4) ***
Diabetes	44 (23.8)	25 (23.4)
Cardiovascular (angina, MI)	34 (18.4)	25 (23.4)
Heart failure	16 (8.7)	10 (9.3)
COPD	2 (1.1)	13 (12.1) ***
Depression	5 (2.7)	27 (25.2) ***
Hospitalization, *n* (%), prior 6 months	50 (27.0)	16 (17.4)^a^
Current prescription medications, mean (SD)	8.04 (2.6)	7.32 (2.5) *
Motivation to deprescribe, mean (SD)	15.4 (4.3)	16.7 (3.9) *
Trust in health care provider, mean (SD)	19.8 (3.4)	21.9 (2.8) ***

*Note:* Motivation to deprescribe (6 items, scale range 6–30); trust (5 items, range 5–25).

^a^ Based on 92 participants.

^b^ College graduate or higher education.

^c^ EQ‐5D‐5L was used to assess each health domain.

* *p* < 0.05.

*** *p* < 0.001.

### 3.3 Motivation to Deprescribe and Trust in Medical Providers, by Location

Interest in deprescribing was high. In the Pittsburgh sample, 73.6% reported discussions with physicians in the past about stopping a medication. Nearly half (47.8%) reported they would be comfortable following a physician's recommendation to stop a medication. In the Kawasaki sample, 48.1% reported having consulted their primary care physician in the past 6 months about changing their medications.

More trust in healthcare providers was associated with lower motivation to deprescribe across both sites (Pearson *r*: −0.35 in Tama [*p* < 0.001], −0.20 in Pittsburgh [*p* < 0.037]). The interaction between location and trust in estimates of motivation to deprescribe was not significant, as indicated by similar slopes across settings in the regression of motivation on trust. A jittered scatterplot of values for motivation to deprescribe by trust is shown in Figure 1, which suggests broad similarity among participants at the two sites. However, while the interaction term in the regression model was not a significant correlate of motivation in this model, power for the test of the interaction was only 0.66.

**FIGURE 1:**
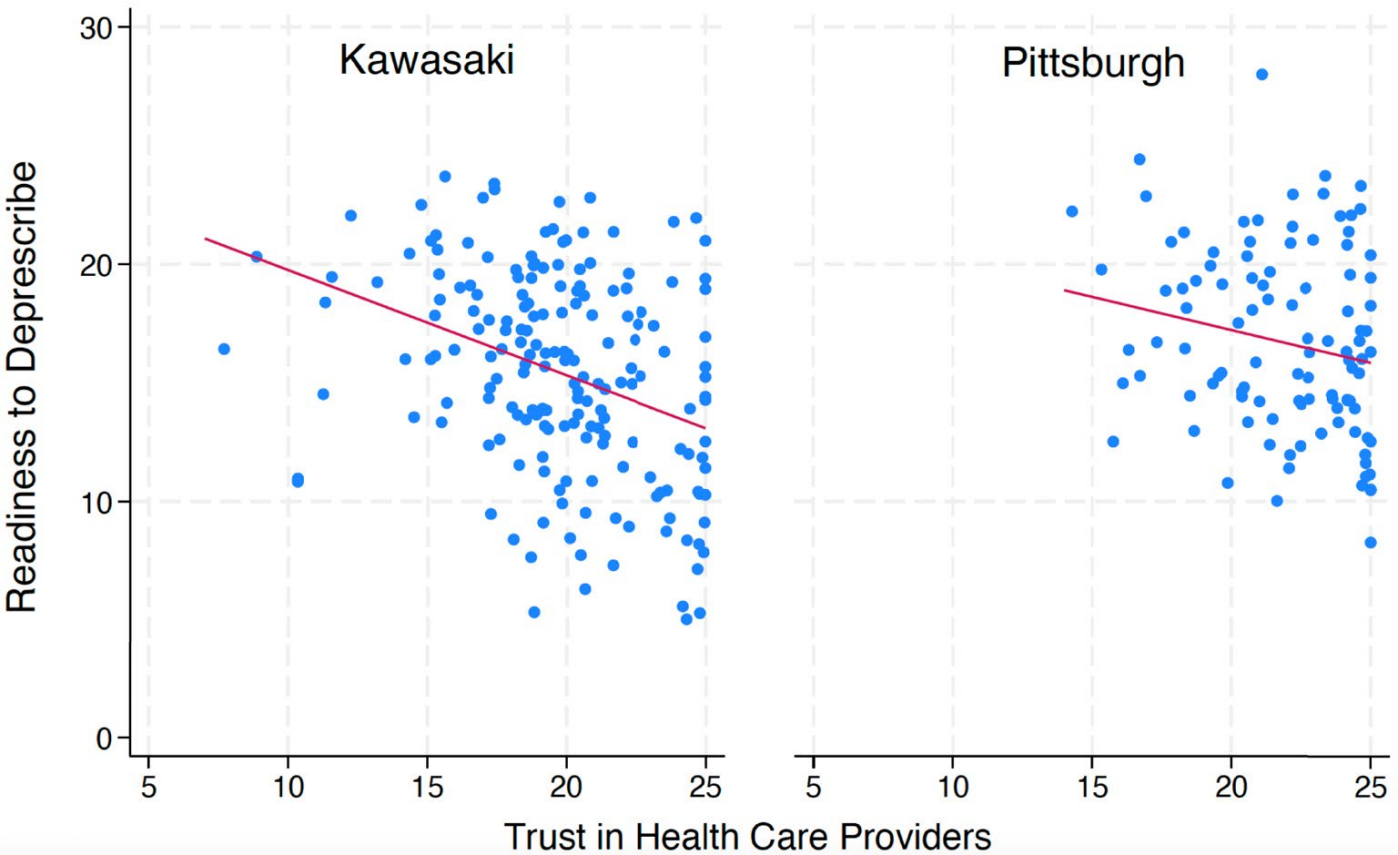
Association between trust in healthcare providers and readiness to deprescribe in Kawasaki and Pittsburgh samples.

In adjusted regression models (Table 2), greater trust in healthcare providers was associated with lower motivation to deprescribe, while a higher number of prescriptions was associated with greater motivation, and location also remained a significant predictor of motivation to deprescribe (+2.45 points in the U.S. sample, *p* < 0.01).

**TABLE 2:** Correlates of motivation to deprescribe.

	*B* (SE)	95% CI
Age category^a^	−0.45 (0.34)	−1.12, 0.23
Male	−0.51 (0.49)	−1.48, 0.46
College graduate^b^	−0.14 (0.57)	−1.3, 0.98
Fair or poor mobility	1.49 (0.74)	0.03, 2.95*
Number of prescription medications	0.32 (0.09)	0.14, 0.50***
Trust in health care provider	−0.40 (0.07)	−0.54, −0.26***
Pittsburgh (vs. Kawasaki)	2.45 (0.60)	1.26, 3.65***

*Note:* *n* = 289, *F* (7, 281) = 8.63, *p* < 0.0001, Adj *R* ^2^ = 0.16.

^a^ Age categories were 65–74, 75–84, and ≥ 85 years.

^b^ College graduate or higher education.

* *p* = 0.046.

*** *p* ≤ 0.001.

Participant motivation to deprescribe (PPoD subscale 1) was significantly higher in the U.S. sample (16.7 ± 3.9) than in the Japanese sample (15.4 ± 4.3), *p* < 0.05. Trust in healthcare providers was also higher in the U.S. sample (21.9 ± 2.8 vs. 19.8 ± 3.4, *p* < 0.001).

## 4 Discussion

This cross‐national study demonstrates a consistent association between trust in healthcare providers and readiness to deprescribe across both the Japanese and U.S. samples. In both settings, greater trust was associated with lower motivation to deprescribe, and the association did not significantly differ by location. Although U.S. participants expressed greater motivation to deprescribe, even after adjusting for differences in health status and medication burden, the pattern of association between trust and motivation was similar across settings.

These findings are consistent with prior studies of attitudes toward deprescribing. A 14‐country European survey reported that patients with higher trust in primary care providers were less likely to name a medication they would like to stop or reduce [7]. The European cross‐national survey also found a greater interest in deprescribing among patients with a higher number of medications, as we note for the Japanese and American samples. In a multivariate model from the European study, only lower satisfaction with medications and greater trust in physicians, but not the number of medications, were associated with deprescribing. Similarly, a Swiss study found an association between willingness to deprescribe and greater trust in primary care providers [16]. The correlation between motivation to deprescribe and trust in medical providers (−0.35 in Kawasaki, −0.20 in Pittsburgh) is similar to a report from a U.S. veterans sample using the PPoD (−0.30) [17]. Other studies, however, have not found as strong a relationship, which may have to do with differences in measures [17, 18, 19, 20].

At the same time, we observed differences in overall levels of readiness of deprescribing and trust in healthcare providers between the two settings. U.S. participants reported higher readiness to deprescribe and greater trust in healthcare providers, even after adjustment for clinical and demographic factors. These results may reflect a variety of factors: differences in lifetime risk of health conditions, in access to health care and diagnostic assessment, in prescribing, and in expectations for health and health care in old age. However, these contextual differences did not appear to modify the relationship between trust and readiness, which remained consistent across settings.

If trust between patients and physicians is associated with readiness to deprescribe, as suggested by our findings, the design of deprescribing interventions may need to consider effects on trust. High trust may be associated with a lower perceived need to question prescribing, potentially limiting opportunities for medication review. Blind trust—when patients leave all decisions to their doctors—may reduce chances to talk openly about medications. In contrast, a more active form of trust allows patients to ask questions and doctors to invite discussion about the care plan. Promoting this form of trust, which combines confidence in the provider with permission to engage, may be essential for successful deprescribing conversations across cultural settings. Healthcare providers may support this active form of trust by using plain language and teach‐back to enhance patients' understanding of medical information, and by using shared decision‐making to ensure that deprescribing decisions reflect patients' goals and preferences [21, 22].

Limitations in this research include differences in setting for ascertaining participants. Participants were recruited from a research registry of patients receiving care from a hospital system in Pittsburgh and from a community hospital and a primary care clinic in Kawasaki, Japan. Thus, observed differences by location may reflect differences in clinical setting, recruitment methods, healthcare access, and participant selection, as well as geographic or cultural context. In addition, low participation in cognitive screening in Pittsburgh may have introduced selection bias related to trust or readiness to deprescribe. Still, a common set of inclusion and exclusion criteria ensured comparability of the samples. There are likely many unmeasured factors that affect motivation to deprescribe or trust in medical providers. However, we assessed health‐related quality of life and diagnosed medical conditions, along with broad sociodemographic indicators, in a common data collection protocol and adjusted for differences in multivariable models. While the health insurance systems of the two countries differ in major ways, participants in the American sample were all Medicare enrollees well connected to university medical services, which lowers the influence of cost as a factor in deprescribing decisions. In addition, statistical power to detect interaction effects was limited, and subtle differences in the association between trust and readiness across settings may not have been fully captured.

In short, although older adults in a U.S. sample demonstrated greater motivation to deprescribe than those in a broadly similar Japanese sample, the association between trust in healthcare providers and readiness to deprescribe was consistent across both settings. These results suggest that trust plays a fundamental role in shaping patients' attitudes toward deprescribing across different cultural contexts. An important question for future research is whether the current effectiveness of deprescribing interventions, in which approximately seven patients need to receive an intervention to get a reduction of one prescribed medication [23] could be enhanced by incorporating strategies that address the role and quality of trust in patient–provider relationships.

An implication of this study is the need for a sensitive approach to promoting trust. Designing interventions that foster a more constructive form of trust, which supports patient confidence while allowing and encouraging open dialog, may be key to sustaining effective deprescribing conversations across cultures. This perspective is also supported by qualitative research suggesting that trust can facilitate meaningful deprescribing discussions when appropriately engaged [24].

## Author Contributions

**Kenya Ie:** conceptualization, methodology, funding acquisition, project administration, writing – original draft, writing – review and editing, formal analysis, investigation, data curation. **Amy M. Linsky:** conceptualization, methodology, investigation, formal analysis, writing – review and editing. **Xinran Liu:** data curation, writing – review and editing, formal analysis. **Melina B. Martin:** writing – review and editing, data curation, investigation. **Rie Oyama:** writing – review and editing, investigation, formal analysis, project administration. **Steven M. Albert:** conceptualization, methodology, data curation, investigation, formal analysis, supervision, funding acquisition, writing – original draft, writing – review and editing.

## Funding

This research was funded by research grants from the Japanese Ministry of Health, Labour and Welfare (grant number: 22K15678), the University of Pittsburgh Claude D. Pepper Older Americans Independence Center (NIH P30 AG024827), research development funds from the University of Pittsburgh School of Public Health, and support from the US Department of Veteran Affairs. The funding sources had no role in the design, methods, participant recruitment, data collection, analysis, or preparation of the manuscript.

## Disclosure

The corresponding author, Dr. Kenya Ie, affirms that all individuals who made significant contributions to this work are appropriately listed as authors or acknowledged, and that written consent was obtained from all contributors named in this section. Dr. Kenya Ie has notified all coauthors of this manuscript submission, subsequent revisions, and the final editorial decision. All author names, order, and affiliations have been confirmed.

## Ethics Statement

The study protocol was approved by the Institutional Ethical Committee of St. Marianna University School of Medicine (No. 5593) and University of Pittsburgh (STUDY23060137).

## Consent

Written informed consent was obtained from all study participants.

## Conflicts of Interest

Dr. Kenya Ie reported grants from the Japanese Ministry of Health, Labour, and Welfare (21GC0801, 25K02859), the Japan Agency for Medical Research and Development (21fk0108486h0001), and the Japan Pharmaceutical Manufacturers Association outside the submitted work, and being a member of the Japan Primary Care Association working group for polypharmacy. Dr. Kenya Ie is an Associate Editor of the Journal of General and Family Medicine; this role had no involvement in the editorial handling of the submitted manuscript. He also reported receiving lecture honoraria from Otsuka Pharmaceutical Factory Inc. and Viatris Pharmaceuticals Japan Inc., outside the submitted work; none of these relationships is considered conflicts of interest with the submitted study. Dr. Amy M. Linsky reported support from the Department of Veterans Affairs, the Center for Health Optimization and Implementation Research (CHOIR), the New England Geriatric Research Education and Clinical Center (GRECC), and VA Boston Healthcare System. She has received support from the US Deprescribing Research Network, which is funded by the US National Institutes of Health. Dr. Xinran Liu reported support from the China Medical Board (CMB) outside the submitted work; this support has no conflict of interest with the submitted study. Dr. Rie Oyama reported support from the Yumi Memorial Foundation for Home Health Care outside the submitted work; this support has no conflict of interest with the submitted study. Dr. Steven M. Albert reported support from the University of Pittsburgh Claude D. Pepper Older Americans Independence Center (NIH P30 AG024827).

## Supporting information


**Table S1:** Commonly used prescribed medications.

## Data Availability

The data that support the findings of this study are available on request from the corresponding author. The data are not publicly available due to privacy or ethical restrictions.
