# Supplementary material for: Association Between Trust in Healthcare Providers and Readiness to Deprescribe in Older Adults: A Cross‐Sectional Comparative Study in the United States and Japan
Source: J Gen Fam Med. 2026 Oct 7;27(6):e70186. doi: 10.1002/jgf2.70186 (PMC13643506; doi:10.1002/jgf2.70186)
Supplement: Supplementary file 1 [file JGF2-27-e70186-s001.docx]

**Supplemental Material. Commonly Used Prescribed Medications**

──────────────────────────────────────
Pittsburgh Sample (n = 107)	　　　　%	Japanese Sample (n = 185)	　　　　%

──────────────────────────────────────
Atorvastatin          	51.4	Amlodipine          	41.1

Levothyroxine        	32.7	Aspirin            	27.6

Amlodipine          	24.3	Lansoprazole        	22.7

Losartan           	21.5	Magnesium oxide      	21.6

Omeprazole         	16.8	Bisoprolol         	20.5

Lisinopril          	15.0	Atorvastatin        	18.9

Albuterol          	13.1	Febuxostat         	17.3

Gabapentin         	10.3	Rosuvastatin        	15.7

—              	—	Esomeprazole        	15.1

—              	—	Pitavastatin         	14.6

—              	—	Eldecalcitol         	13.5

—              	—	Metformin          	13.0

—              	—	Vonoprazan         	11.4

—              	—	Nifedipine          	11.4

—              	—	Furosemide         	10.3

—              	—	Telmisartan         	10.3

──────────────────────────────────────
Values are percentages of participants taking each medication.

Medications listed were those taken by more than 10% of participants in each sample.

Only prescribed medications were included; over-the-counter (OTC) and non-prescribed medications were excluded.
